# The Effect of Intraoperative Patient Positioning on the Success of Intertrochanteric Fracture Surgery in Older Patients

**DOI:** 10.3390/medicina60040646

**Published:** 2024-04-18

**Authors:** Onur Kaya, Buğra Kundakçı, Cem Önder, Vahap Kurt, Emre Atmaca, Fatih Tunç

**Affiliations:** 1 Department of Orthopaedic and Traumatology, NCR Private Hospital Mücahitler, Gazi Muhtar Paşa Blv. No:56, 27090 Şehitkamil, Gaziantep, Türkiye; 2 Department of Orthopaedic and Traumatology, Faculty of Medicine, Cukurova University, 01330 Sarıçam, Adana, Türkiye; bugrakundakci@hotmail.com (B.K.); tuncfatih93@gmail.com (F.T.); 3 Department of Orthopaedic and Traumatology, Abdulkadir Yuksel State Hospital, 27100 Şahinbey, Gaziantep, Türkiye; cmndr92@gmail.com (C.Ö.); vahapkurt@hotmail.com (V.K.); eatmaca07@gmail.com (E.A.)

**Keywords:** PFN, intertrochanteric fracture, traction table, supine hemilithotomy, lateral decubitus

## Abstract

*Background and Objectives:* The incidence of hip fractures in people of advanced ages is increasing due to our aging society. Patient positioning for the intertrochanteric fractures of the femur can be performed in various ways. The aim of this study is to clinically and radiologically compare the use of the supine hemilithotomy position, the lateral decubitus position, and the traction table when performing proximal femoral nail (PFN) surgery for femoral intertrochanteric fractures in the geriatric age group. *Materials and Methods:* A total of 170 elderly patients with femoral intertrochanteric fractures were included in this cross-sectional study. The patients were divided into three groups (the supine hemilithotomy group, the lateral decubitus group, and the fracture table group). For the postoperative period, complications, length of stay in the intensive care unit, and length of stay in hospital were examined, while in postoperative radiographs, tip–apex distances (TADs), collodiaphyseal angles (CDAs), and Cleveland–Bosworth quadrants were examined to evaluate the placement of the lag screw in the femoral head. The quality of fracture reduction was evaluated according to the modified Baumgaertner criteria. *Results:* The mean age of the patients was 77.8 ± 8.8; 57.6% of patients were female. According to the modified Baumgaertner criteria, it was determined that patients with ‘poor’ reduction quality had an approximately ten times higher risk of cut-out than those with ‘good’ reduction quality (OR = 10.111, *p* = 0.002, 95% confidence interval; 2.313–44.207). The operative time for patients in the fracture table group was longer than that of the other groups Additionally, the CDA in the supine hemilithotomy position group was longer. *Conclusions:* Although PFN surgery using the traction table is longer in terms of surgical time compared to surgery performed in the lateral decubitus position and the supine hemilitotomy position, it is advantageous in terms of better TAD and CDA values and lower complication rates.

## 1. Introduction

Geriatric hip fractures are considered a significant orthopedic problem around the world [1]. The incidence of hip fractures in people of advanced ages is increasing due to our aging society [2]. Hip fractures pose a serious threat in terms of morbidity and mortality, and early detection and prompt ambulation are crucial for these patients [3]. Prolonged immobilization in the postoperative period can result in pneumonia, heart failure, delirium, muscle weakness, and even death in some patients [4]. 

It is crucial that the patient’s positioning during surgery does not complicate imaging and surgical exposure while facilitating fracture reduction. For trochanteric region fractures of the femur, patient positioning can be achieved in three ways: supine on the radiolucent table, lateral decubitus on the radiolucent table, and supine on the fracture table [5]. Each of these positioning methods has its own set of advantages and disadvantages.

While the traction table provides axial traction on the legs, it hinders trunk traction due to a perineal post. Notable advantages include the fact that an assistant for traction is not required, its ability to be performed by a single surgeon, and the ease of visualization. However, significant disadvantages include the risk of perineal nerve damage, inconvenience for obese patients, and the high cost associated with it [4,6].

The significant advantages of surgery performed in the lateral decubitus position include its ability to facilitate easy access to the starting point, particularly in obese patients, and its applicability on universally used surgical tables. Additionally, during open surgery, it is more feasible to mitigate the effects of muscles that exacerbate deformity and facilitate fracture reduction. However, notable disadvantages include the requirement for more than one assistant, difficulties in imaging, and the inability to compare rotation with the other leg [4].

Performing fracture reduction in the supine hemilithotomy position on a radiolucent table with manual traction enables a reduction in the fracture while minimizing complications associated with continuous traction and facilitating easy radiological imaging. However, important disadvantages include the requirement for a longitudinally adjustable table and the necessity of having two or more assistants [7].

The aim of this study was to compare the clinical and radiological results of proximal femoral nail (PFN) surgery for intertrochanteric femoral fractures in the geriatric population in three groups not previously compared in the literature (the supine hemilithotomy position group, the lateral decubitus position group, and the traction table group).

## 2. Materials and Methods

Data were extracted from the hospital’s electronic data system. Patients were included if they were aged 65 years or older with acute AO/OTA (AO Foundation/Orthopedic Trauma Association); had classification types 31A1, 31A2, and 31A3; had femoral intertrochanteric fractures; and underwent closed reduction and osteosynthesis with short PFN surgery between 2020 and 2022.

The exclusion criteria were as follows: pathologic fractures, open fractures, the utilization of open methods during surgery, the use of additional implants, infection in the incision area, blood diseases, polytrauma, and severe acute or chronic inflammatory diseases. The patients who underwent PFN surgery conducted by experienced surgeons were categorized into three groups according to the surgical position: supine hemilithotomy, traction table, and lateral decubitus. The preference for different surgical positions was informed by the variation in methods familiar to surgeons trained in different clinics. Patients with accessible complete medical records were included in the study. 

**Figure 1:**
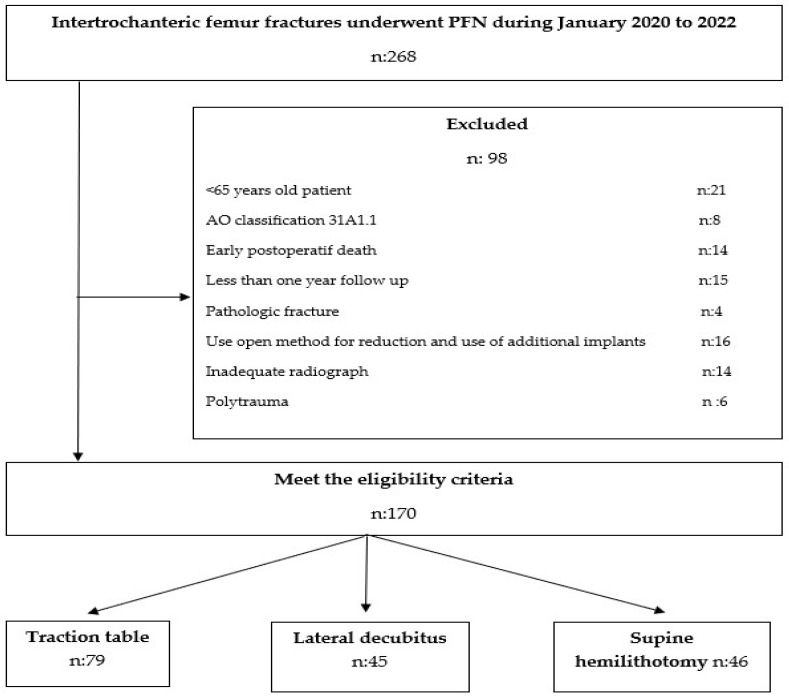
Patient selection flow chart.

Patients were followed up with for a minimum of one year. Those who died within one year and patients with less than one year of follow-up were excluded from the study. Our study was carried out with 170 patients selected based on the specified inclusion and exclusion criteria (Figure 1).

Fractures were categorized according to the AO/OTA classification. According to this classification, 31A1.2 and 31A1.3 are classified as stable, while 31A2.1, 31A2.2, 31A2.3, 31A3.1, 31A.2, and 31A3.3 are classified as unstable [8].

### 2.1. Clinical and Radiological Assessment 

**Figure 2:**
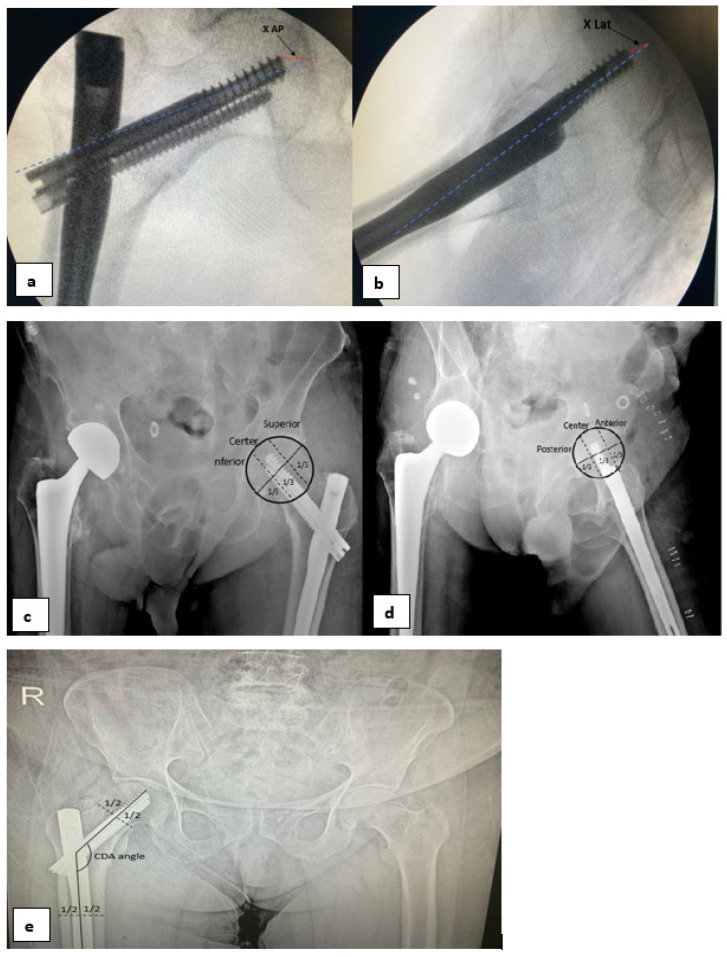
Methods of measuring TAD values. TAD = [Xap × (Dtrue/Dap)] + (Xlat × (D true/Dlat)] (Dtrue = known diameter of the lag screw) (**a**,**b**). Determination of quadrant in the anteroposterior view and lateral view (**c**,**d**). Measurement of the collodiaphyseal angle (CDA) (**e**).

**Figure 3:**
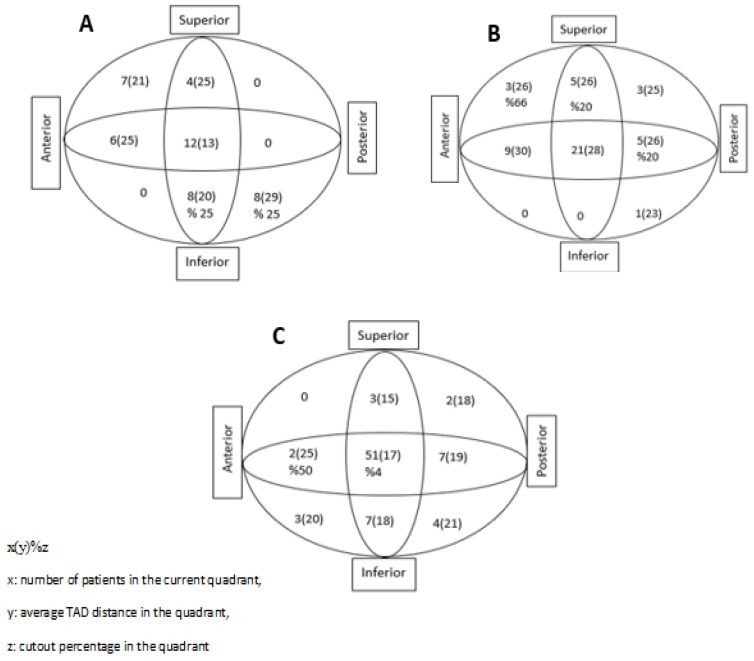
Percentage of cut-out complications and mean tip–apex distances (millimeters) for each quadrant. (**A**). Lateral decubitus position; (**B**) supine hemilithotomy position; and (**C**) traction table.

Preoperative data on the patient’s age, gender, and preoperative comorbidities (diabetes, hypertension, coronary artery disease, heart failure, renal failure, asthma, COPD—chronic obstructive pulmonary disease, dementia, and cerebrovascular disease) were collected. Additionally, the American Society of Anesthesiologists Physical Status Classification System (ASA) score and the number of days between the occurrence of the fracture and surgery were examined. Perioperative data included the duration of surgery from the beginning of anesthesia until the patient left the operating room. For the postoperative period, postoperative complications, length of stay in the hospital, and length of stay in the intensive care unit were examined, while in postoperative radiographs, tip–apex distances (TADs), collodiaphyseal angles (CDAs) (Figure 2), and Cleveland–Bosworth quadrants were examined to evaluate the placement of the lag screw in the femoral head (Figure 3). Two orthopedists performed radiological measurements and categorization using anteroposterior and lateral pelvic radiographs. In addition, screw cut-out evaluations were made during the follow-ups. The quality of fracture reduction was evaluated according to the modified Baumgaertner criteria (Table 1). The adequacy of reduction was classified as good, acceptable, or poor [9].

### 2.2. Surgical Preparation

Surgical procedures were conducted under spinal anesthesia, with orthopedic surgeons in the same team, each possessing at least five years of specialized experience. One hour before surgery, all patients received 1 g of cefuroxime sodium parenterally. Low-molecular-weight heparin was administered 12 h before surgery for prophylaxis against venous thromboembolism and resumed at the twelfth postoperative hour. Upon positioning the patients on the operating table, the surgical site was routinely cleansed with povidone–iodine, and sterile draping was uniformly performed in all groups.

### 2.3. Nail Type

We used a PFN system (ASES® Medikal, Gaziantep, Turkey) with a trapezoidal cross-section and collodiaphyseal angle of a 130°, and provided compression of the head/neck up to 15 mm using an 11 mm lag screw and a 7 mm screw.

### 2.4. Supine Hemilithotomy Position

**Figure 4:**
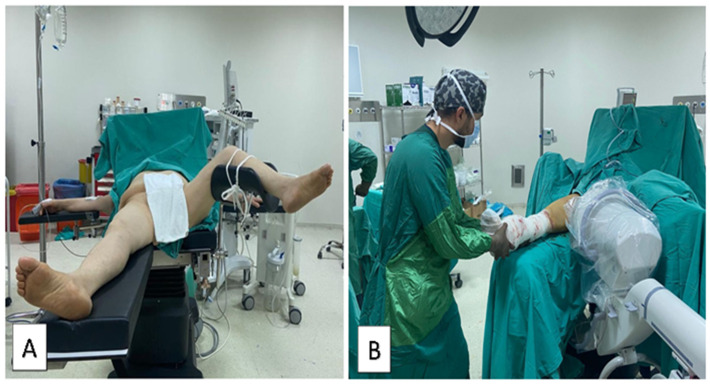
(**A**) Intraoperative view and (**B**) C-arm setup of intramedullary nailing facilitated by manual traction applied by a surgical assistant in the supine hemilithotomy position.

Patients were positioned supine on the operating table. Subsequently, a folded sheet in the form of a roll was placed under the fractured side and was elevated. The unaffected leg was then positioned in the lithotomy apparatus, flexed, and abducted. Following this, the leg apparatus on the unaffected side of the operating table was removed, and the upper torso was laterally adjusted to ensure pelvis lateralization. The scope was then positioned to enter through the middle. The surgical assistant achieved fracture reduction by applying longitudinal traction and rotation. Fracture reduction and implant placement were assessed using C-arm fluoroscopy imaging (Figure 4).

### 2.5. Using the Traction Table

**Figure 5:**
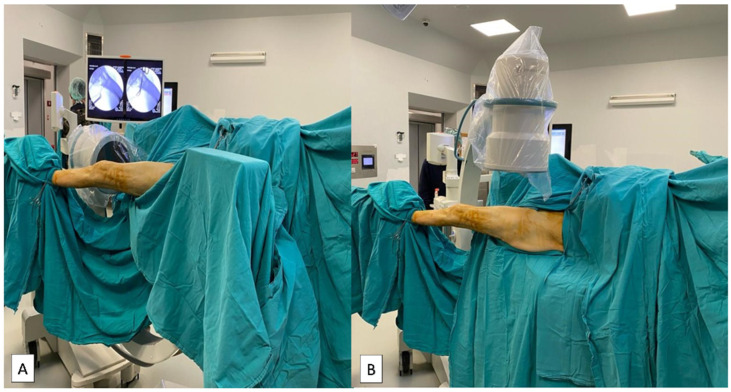
(**A**) C-arm setup and antero-posterior images and (**B**) lateral images of intramedullary nailing facilitated by the traction table.

Patients were anesthetized on the stretcher and subsequently transferred to the traction table. One leg was positioned in the lithotomy apparatus, and the foot on the fracture side was placed in a boot and securely fastened. To prevent the patient from sliding downward, a perineal post was utilized. A sheet was rolled up under the affected hip and elevated while the upper torso was laterally adjusted. The scope was placed between the legs and appropriately positioned. Fracture reduction was accomplished using the traction apparatus and hinges on the sole of the boot, allowing rotation. Once the desired reduction was achieved, the apparatus was locked in place (Figure 5).

### 2.6. Lateral Decubitus Position

**Figure 6:**
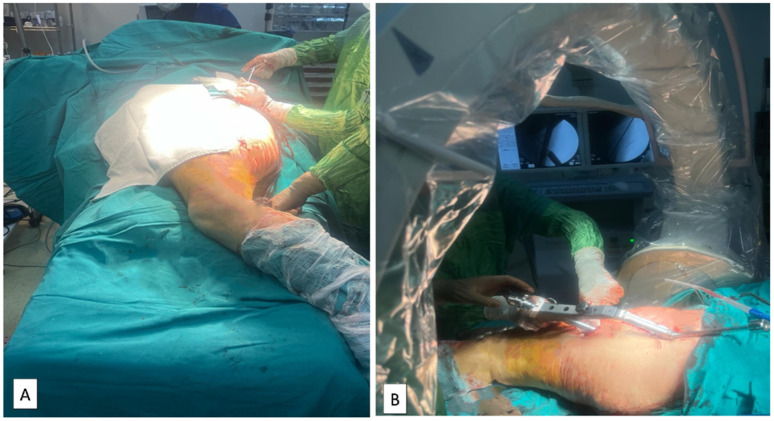
(**A**) Intraoperative view and (**B**) C-arm setup of intramedullary nailing facilitated by the lateral decubitus position.

Following anesthesia administration on the operating table, the patient was gently turned onto their side, with the fractured extremity positioned superiorly. Supports were then strategically placed posterior to the sacrum and anterior to the abdomen. The fluoroscope was positioned opposite to the surgeon. Fracture reduction was accomplished while the assistant applied longitudinal traction and rotation (Figure 6).

### 2.7. Surgical Technique 

Once all preparations were completed on the operating table, an incision was made from the proximal lateral aspect of the greater trochanter, guided by anterior–posterior and lateral radiographs under C-arm fluoroscopy control. Initial proximal reaming was carried out, with no diaphyseal reaming performed in any patient. Subsequently, a short proximal femur nail (10–13 mm wide and 170–240 mm long) was initially secured with a longitudinal lag screw and then with a compression screw. A distal locking screw was then statically fixed. Following a meticulous check of the implant’s position under fluoroscopy control, the procedure concluded with suturing and dressing.

### 2.8. Postoperative Management

Prophylactic 4 × 1 g cefazolin sodium was given parenterally to all patients in the first 24 h after surgery. All patients were given low-molecular-weight heparin for the first three weeks. On the first day following surgery, all patients were mobilized with partial weight bearing, a walker was utilized, and quadriceps exercises commenced.

Our study was conducted retrospectively after obtaining ethics committee approval from the Gaziantep University Ethics Committee (2023/358).

### 2.9. Statistical Analysis

Statistical analysis of the data was conducted using the SPSS 25.0 software package. Categorical measurements were presented as numbers and percentages, while continuous measurements were summarized as means and standard deviations (medians and minimum–maximum values where necessary). A comparison of categorical variables was performed using the chi-square test or Fisher’s test statistics. For a comparison of continuous measurements between groups, the distributions were examined. One-way analysis of variance (ANOVA) was applied to variables with a parametric distribution, and the Kruskal–Wallis test was employed for variables without a parametric distribution.

## 3. Results

The mean age of the patients was 77.8 ± 8.8; 42.4% of patients were male and 57.6% were female. Regarding positioning, 26.5% of the patients were placed in the lateral decubitus position group, 27.1% in the supine position group, and 46.5% in the traction table group (Table 2). Complications were predominantly observed in patients who underwent surgery in the lateral decubitus position, and these occurrences were statistically significantly higher compared to the other groups (Table 3).

The duration of surgery for patients in the traction table group was determined to be longer than that in the other groups. The collodiaphyseal angle values for individuals in the supine position group were lower compared to those in the other groups, and the tip–apex distance for patients in the supine position group was longer than that in the other groups (Table 4). When evaluating patients based on fracture stability, irrespective of the groups, we noted longer TAD measurements in unstable fractures. Additionally, there was a tendency towards varus in unstable fractures (Table 5).

As a result of logistic regression analysis, it was concluded that except for the “Modified Baumgaertner” variable, all other independent variables included in the model were not significant in terms of cut-out risk in all the groups. According to the modified Baumgaertner criteria, it was determined that patients with ‘poor’ reduction quality had an approximately 10 times higher risk of cut-out than those with ‘good’ reduction quality (OR = 10.111, *p* = 0.002, 95% confidence interval: 2.313–44.207) (Table 6). In the patients with cut-out complications, screw placement was noted in the inferior posterior quadrant in two patients and in the inferior central quadrant in two patients who were operated on in the lateral decubitus position. Screw placement was noted in the superior anterior quadrant in two patients who were operated on in the supine hemilithotomy position, in the central superior quadrant in one patient, and in the central posterior quadrant in one patient. In patients who underwent operation on the traction table, screw placement was detected in one patient in the central anterior quadrant and in two patients in the central quadrant (Figure 3).

Other complications, excluding cut-out, were most common in patients who underwent surgery in the lateral decubitus position. Prolonged wound discharge was observed in five patients as a complication, and, in one of these cases, washing and debridement were initially performed under operating room conditions. No infection was observed in the long term. Deep vein thrombosis (DVT) was observed in two patients, while pneumonia occurred in one patient, atelectasis in one patient, and delirium in two patients.

Pulmonary embolism was observed in two patients, while DVT was seen in one patient in the supin hemilithotomy position. Additionally, cellulitis was observed around the wound site in one patient and was successfully treated with antibiotic therapy.

## 4. Discussion

While the reduction in and fixation of unstable intertrochanteric fractures pose challenges, new strategies and implants are continuously being developed to achieve successful fixation and prevent complications, such as inadequate fixation necessitating secondary surgery [10,11,12]. In unstable intertrochanteric fractures, there is no conclusive evidence indicating that the type of intramedullary implant and surgical position reduces the failure rate. However, emphasis has been placed on the significance of surgical skills, including intramedullary nailing reduction, the appropriate placement of the intramedullary nail, and the correct positioning of the lag screw [13]. Furthermore, reduction quality is considered one of the most crucial parameters for achieving favorable clinical and radiological outcomes in contemporary surgery [14,15]. In our study, the quality of reduction was assessed using a classification divided into three grades, based on a modified version of the method developed by Baumgaertner et al. [9]. In the logistic regression analysis comparing patients with and without cut-out regardless of the groups, it was seen that poor reduction quality increased the probability of cut-out tenfold (*p* = 0.002, OR 10.11, logistic regression analysis).

In the logistic regression analysis, by comparing patients with and without cut-out across all groups, it was observed that poor reduction quality increased the probability of cut-out tenfold (*p* = 0.002, OR 10.11, logistic regression analysis). It was reported that the most crucial complication seen after PFN surgery for unstable femoral intertrochanteric fractures is cut-out, and its incidence was found to range between 2% and 3.5%. Cut-out results from the collapse of the femur neck shaft angle into a varus position, leading to the extrusion of the screw from the femoral head [16]. Although cut-out was observed in 6.4% of all patients in our study, no significant difference was found between the groups.

After surgery for intertrochanteric fractures, a CDA < 125° is considered to be varus, 125–135° is neutral, and >135° is valgus. [17,18]. In our study, the mean CDAs of patients were 129.3° across all groups. When comparing the CDAs between groups, it was observed that they were better in the traction table group than in the other groups (*p* = 0.001, chi-squared test). However, no significant relationship was found between cut-out and CDAs, regardless of the groups.

Several significant studies have demonstrated that a TAD of <25 mm reduces the risk of cut-out [16,19]. In this study, the mean TAD of all patients was 21.9 mm. The results were significantly better in those who underwent surgery on the traction table compared to those in other groups (*p* = 0.0001, chi-squared test), while the TAD was measured longer in the supine hemilithotomy position. Additionally, following surgery for unstable fractures, TAD measurements were significantly longer than those for stable fractures. However, regardless of the groups, no significant relationship was found between the TAD and cut-out values. In studies conducted for unstable intertrochanteric fractures, John et al. and Çepni et al. did not find a significant relationship between the TAD and cut-out values and predicted that the TAD alone would not be sufficient to determine the risk of cut-out [20,21]. In their study, however, Fuji et al. suggested that a TAD > 20 mm alone was a prognostic factor for cut-out [22]. Some clinical studies emphasize that even if a TAD is >25 mm, the inferior–central placement of the lag screw may be more stable than central–central placement. They also suggest that a cut-off value of 25 mm should be adjusted by considering the differences in femoral head geometry from patient to patient [23].

When examining the Cleveland–Bosworth quadrants, the femoral neck was divided into three regions: superior, center, and inferior in the coronal plane and anterior, center, and posterior in the sagittal plane. Nine quadrants were defined after the intersection of these regions. In the study by Hwang et al., the center–center or inferior–center quadrants were recommended [24]. Karapınar et al. found the center–center, inferior–center, and inferior–posterior quadrants to be safe [25]. De Bruijn et al. identified the inferior–anterior and inferior–posterior quadrants as safe [26]. In our study, upon performing an examination within the groups and independently of the groups, no relationship was found between the quadrants alone and the cut-out rate.

The prolongation of surgical setup and the longer duration of surgical procedures due to anesthesia in these patients increase complications and mortality [27]. When comparing all three positions in our study, it can be observed that the duration of surgery was longer when performed on the traction table. However, the length of this period did not make a significant difference in terms of developing complications. The prolonged preparation phase is thought to be the reason for the longer duration of surgery observed in the traction table group.

In our study, it was observed that the surgical time for patients operated on in the lateral decubitus position was significantly shorter compared to that in the other groups (*p*=0.0001, chi-squared test). However, the duration of surgery in the group who underwent surgery on the traction table was significantly longer than in the other groups. In a study by Şahin et al., comparing manual traction and the traction table, it was determined that the surgery time and total anesthesia time were lower in the manual traction group, whereas the number of assistants required was lower in the traction table group [28]. In a study comparing surgery performed with the traction table and surgery performed in the lateral decubitus position, Sadeq et al. emphasized that the surgical time was shorter when performed in the lateral decubitus position, but the quality of reduction was better on the traction table; thus, these results are similar to those in our study [29]. In their study, Kakumanu et al. highlighted that the lateral decubitus position makes it easier to find the nail entry point and is a preferable method, especially for obese patients [30].

In a study comparing the supine hemilithotomy position with the traction table, it was observed that there was no significant clinical or radiological difference between these two positions, except for the duration of surgery [31].

In our study, it was found that patients in the group that underwent surgery on the traction table were older and had more comorbidities than individuals in the other groups. The time from fracture to surgery for patients in the traction table group was longer compared to that in the other groups due to the consultations required to solve cardiac and internal problems for anesthesia in the preoperative period. A recent study has emphasized that cardiology consultation and echocardiogram in the preoperative period for hip fractures in the geriatric age group are both expensive and unnecessary [32].

## 5. Conclusions

In conclusion, although PFN surgery performed using a traction table took longer in terms of surgical time compared to surgery performed in the lateral decubitus position and supine hemilithotomy position, the fact that the TAD and CDA values were better and the complication rate was lower is important. It was observed that the duration of surgery was shorter, but the complication rate was higher when performed in the lateral decubitus position. When the results were evaluated independently of the groups, we considered that successful fracture reduction is the most important way to prevent cut-out. Although surgical duration in the traction table group is longer compared to that in other groups, we believe it is superior to other surgical positions due to its better outcomes. We believe that our study will serve as a guide for future prospective studies with a larger numbers of patients.

### Limitations 

There are some limited aspects of the study. First of all, the study was planned retrospectively. In addition, bone quality and body mass index were not included. Moreover, our study is also limited in the sense that the male–female ratio was not equal, all fractures were unstable, and all surgical interventions were not performed by the same surgeon.

## Figures and Tables

**Table 1:** The assessment of reduction quality according to the modified Baumgaertner criteria.

Modified Baumgaertner Criteria		
1. Alignment	2. Displacement of Fragments	Reduction Quality
(a) Anteroposterior view: Normal or slight valgus neck–shaft angle (b) Lateral view: Less than 20° of angulation	(a) >80% overlap (b) <5 mm shortening	Good: Both criteria met Acceptable: Only one criterion met Poor: Neither criterion met

**Table 2:** Demographic distribution of patients.

		N	Mean ± SD	Median	Min-Max
Age		170	77.8 ± 8.8	79	65–99
ASA score		170	2.8 ± 0.7	3	1–4
Time between fracture and surgery (days)		170	3.1 ± 2.3	2	1–10
Duration of surgery (minutes)		170	110.6 ± 45.2	105	35–285
Duration of intensive care unit stay (days)		170	2.4 ± 3.8	2	0–30
Duration of hospital stay (days)		170	8.3 ± 4.5	7	0–31
Collodiaphyseal angle		170	129.3 ± 6.7	130	110–146
TAD (mm)		170	21.9 ± 9.1	20	9–74
Cut-out (days)		11	39.5 ± 27.3	30	25–120
Gender	Male	72(42.4%)			
	Female	98(57.6%)			
Groups	Lateral decubitus	45(26.5%)			
	Supine hemilithotomy	46(27.1%)			
	Traction table	79(46.5%)			
AO Stable	31A1.2	22(12.9%)			
	31A1.3	34(20.5%)			
AO Unstable	31A2.1	50(29.4%)			
	31A2.2	14(8.2%)			
	31A2.3	37(21.7%)			
	31A3.1	7(4.1%)			
	31A3.3	5(2.9%)			

**Table 3:** Findings for group comparisons based on the demographic characteristics of patients.

Variables	Category	Lateral Decubitus		Supine Hemilithotomy		Traction Table		*p*
		N (%)		N (%)		N (%)		
Gender	Male	26 (57.8)		20 (43.5)		26 (32.9)		0.026 *
	Female	19 (42.2)		26 (56.5)		53 (67.1)		
Comorbidities	No	18 (40)		7 (15.2)		4 (5.1)		<0.001 *
	Yes	27 (60)		39 (84.8)		75 (94.9)		
Number of comorbidities	1 comorbidity	8 (29.6)		15 (38.5)		24 (32)		0.709
	2 and more comorbidities	19 (70.4)		24 (61.5)		51 (68)		
Modified Baumgaertner	Good	18 (40)		30 (65.2)		46 (58.2)		0.054
	Acceptable	17 (37.8)		14 (30.4)		21 (26.6)		
	Poor	10 (22.2)		2 (4.3)		12 (15.2)		
Cut-out	No	41 (91.1)		42 (91.3)		76 (96.2)		0.418
	Yes	4 (8.9)		4 (8.7)		3 (3.8)		
Complications	No	34	75.6	42	91.3	79	100	<0.001 *
	Yes	11	24.4	4	8.7	0	0	

* *p* < 0.05; chi-square test.

**Table 4:** Descriptive statistics and comparison results for the demographic and general characteristics of groups.

	Traction Table (N = 79)	Supine Hemilithotomy (N = 46)	Lateral Decubitus (N = 45)	*p*	p_TT&SH_	p_TT&LD_	p_SH&LD_
Age	78.5 ± 8.4	75.6 ± 8.2	76.9 ± 9.4	0.059		-	-
ASA score	2.7 ± 0.6	3.1 ± 0.8	2.7 ± 0.6	0.006	0.002	0.392	0.022
Time between fracture and surgery (days)	4 (1–10)	2 (0–5)	1 (1–9)	0.0001	0.0001	0.0001	0.935
Duration of surgery (minutes)	135 (45–285)	95 (40–155)	70 (35–180)	0.0001	0.0001	0.0001	0.0001
Duration of intensive care unit stay (days)	0 (0–8)	3 (0–30)	2 (0–23)	0.0001	0.0001	0.0001	0.803
Duration of hospital stay (days)	8 (3–24)	7 (7–31)	6 (3–26)	0.680	-	-	-
Collodiaphyseal angle	129.5 ± 4.9	126.6 ± 5.7	133 (111–146)	0.001	0.001	0.012	0.001
TAD (mm)	17 (9–30)	26 (16–74)	18.3 (9.5–49.1)	0.0001	0.0001	0.322	0.0001

**Table 5:** The measurement of CDAs, TADs, and cut-out ratios according to fracture stability, and assessment was made according to the modified Baumgaertner criteria.

	AO Stable (n = 56)	AO Unstable (n = 114)	*p*
Collodiaphyseal angle	131.2 ± 5.2	128.4 ± 7.2	0.010
TAD	17.4 ± 5.4	24.1 ± 9.7	0.0001
Cut-out	4 (7.1)	7 (6.1)	0.753
Modified Baumgaertner			
Good	36 (64.3)	58 (50.9)	0.239
Poor	7 (12.5)	17 (14.9)	
Acceptable	13 (23.2)	39 (34.2)	

**Table 6:** Findings based on the modeling of participants’ cut-out situations according to their demographic characteristics.

		Beta	SE of Beta	Odds Ratio	95% CA	*p*
Age		−0.020	0.036	0.980	0.914–1.052	0.582
Gender		−1.264	0.798	0.283	0.059–1.350	0.113
ASA score		0.425	0.466	1.530	0.613–3.816	0.362
Comorbidities (Yes/No)		0.083	0.810	1.086	0.222–5.312	0.918
Cleveland–Bosworth quadrant		0.048	0.156	1.049	0.773–1.425	0.759
Modified Baumgaertner	Ref: Good					0.003 *
	Acceptable	0.193	0.930	1.213	0.196–7.505	0.835
	Poor	2.314	0.753	10.111	2.313–44.207	0.002
Collodiaphyseal angle		0.085	0.049	1.089	0.988–1.199	0.085
Tip–apex distance		0.022	0.030	1.022	0.964–1.083	0.470

* *p* < 0.05; logistic regression analysis.

## Data Availability

Data are contained within the article.
